# Differences in characteristics between patients ≥ 65 and < 65 years of age with orthopaedic injuries after severe trauma

**DOI:** 10.1186/s13049-022-01038-w

**Published:** 2022-09-24

**Authors:** Tora Julie Slørdal, Guttorm Brattebø, Thomas Geisner, Målfrid Holen Kristoffersen

**Affiliations:** 1 Faculty of Medicine, University of Bergen, Haukelandsveien 28, 5009 Bergen, Norway; 2 Department of Anaesthesia and Intensive Care, Haukeland University Hospital, Bergen, Norway; 3 Western Norway Trauma Centre, Haukeland University Hospital, Bergen, Norway; 4 Department of Orthopaedic Surgery, Haukeland University Hospital, Bergen, Norway

**Keywords:** Major trauma, Multiple trauma, Polytrauma, Orthopaedic injuries, Older adults, Traumatic, Epidemiology, Trauma registry, Injury severity score, Mortality

## Abstract

**Aim:**

Many trauma patients have associated orthopaedic injuries at admission. The existing literature regarding orthopaedic trauma often focuses on single injuries, but there is a paucity of information that gives an overview of this group of patients. Our aim was to describe the differences in characteristics between polytrauma patients ≥ 65 and < 65 years of age suffering orthopaedic injuries.

**Methods:**

Patients registered in the Norwegian Trauma Registry (NTR) with an injury severity score (ISS) > 15 and orthopaedic injuries, who were admitted to Haukeland University Hospital in 2016–2018, were included. Data retrieved from the patients’ hospital records and NTR were analysed. The patients were divided into two groups based on age.

**Results:**

The study comprised 175 patients, of which 128 (73%) and 47 (27%) were aged < 65 (Group 1) and ≥ 65 years (Group 2), respectively. The ISS and the new injury severity score (NISS) were similar in both groups. The dominating injury mechanism was traffic-related and thoracic injury was the most common location of main injury in both groups. The groups suffered a similar number of orthopaedic injuries. A significantly higher proportion of Group 1 underwent operative treatment for their orthopaedic injuries than in Group 2 (74% vs. 53%). The mortality in Group 2 was significantly higher than that in Group 1 (15% vs. 3%). In Group 2 most deaths were related to traffic injuries (71%). High energy falls and traffic-related incidents caused the same number of deaths in Group 1. In Group 1 abdominal injuries resulted in most deaths, while head injuries was the primary reason for deaths in Group 2.

**Conclusions:**

Although the ISS and NISS were similar, mortality was significantly higher among patients aged ≥ 65 years compared to patients < 65 years of age. The younger age group underwent more frequently surgery for orthopaedic injuries than the elderly. There may be multiple reasons for this difference, but our study does not have sufficient data to draw any conclusions. Future studies may provide a deeper understanding of what causes treatment variation between age groups, which would hopefully help to further develop strategies to improve outcome for the elderly polytrauma patient.

## Background

Severely injured patients comprise a major challenge and require close inter-professional collaboration and comprehensive care. It has been reported that nearly half of the patients with major trauma suffer one or more musculoskeletal injuries [1]. Such injuries may have impact on long-term outcomes [2]. These patients often undergo multiple surgical procedures for their orthopaedic injuries. Moreover, the subsequent rehabilitation of orthopaedic injuries frequently requires vast resources [2, 3].

The existing literature regarding orthopaedic trauma often focuses on single injuries, whereas studies describing the characteristics of the patients sustaining these injuries are scarce [4]. Information about the extent, localisation, and outcomes of trauma-related injuries may affect the treatment of these patients.

Trauma registries are useful tools for quality assessment of trauma systems. Analysing registry data may provide valuable information about the patient group and the treatment given, which in turn can be used to evaluate and improve e.g. treatment protocols [5].

Previously, traumatic injuries were primarily affecting young men, but as the population ages, the number of older trauma patients is increasing [6, 7]. When exposed to traumatic injury, advanced age is often a liability associated with increased mortality and morbidity [8]. The elderly are characterized by limited physiologic reserves, high frequency of underlying diseases and polypharmacy, which increases the risk of post-injury complications and poor functional outcome [9]. Data suggest that despite their vulnerabilities, the elderly frequently receive inferior trauma care when compared to younger patients [9]. Improving patient safety and reducing the risk of increased morbidity and mortality for vulnerable groups like the elderly, are important aspects of trauma care development. Hence, there is need for particular focus on the epidemiology of geriatric trauma patients.

## Aim

The purpose of this study was to describe differences in characteristics between polytrauma patients ≥ 65 and < 65 years of age, suffering orthopaedic injuries and hospitalised in a major university hospital in Norway during the period 2016–2018.

## Methods

The Norwegian Trauma Registry (NTR) is a national medical quality registry established in 2006, with web-based registrations starting in 2015. NTR´s main objective is to monitor, assess, and improve quality of trauma care. It is a collective endeavour, where 38 trauma-receiving hospitals supply information to a national database [10]. Certified personnel collect data from injury to rehabilitation, in accordance with the Utstein template [11]; classify injuries according to the abbreviated injury scale (AIS) [12]; and calculate the injury severity score (ISS) [13]and new injury severity score (NISS) [14].

NTR includes all patients admitted with trauma team activation (TTA) upon arrival at the emergency department irrespective of severity scores (ISS/NISS), as well as individuals admitted with penetrating injuries, severe head injuries (AIS ≥ 3) and NISS > 12 in the absence of TTA. Patients who succumb on the site of injury or during transportation are also included if prehospital resources were activated. However, patients with solitary chronic subdural hematoma and bodily harm without concomitant trauma are not included in the NTR [7].

Annually, the NTR retrieves information on approximately 8,000 trauma patients, of whom 1:8 have an ISS > 15. Approximately 12% of the patients with an ISS > 15 in Norway are admitted to Haukeland University Hospital (HUH) in Bergen, one of four trauma centres (TCs) in Norway and the regional TC in the western health region of Norway (Rogaland and Vestland) [10, 15]. The population in the 45,000-km2 area is approximately 1.1 million, a third of which live in the two major regional centres Bergen and Stavanger [16].

All patients registered in HUH´s local NTR database with an ISS > 15 during the period 2016–2018 were included in the study. ISS > 15 is the most common threshold when defining major trauma [17]. Exclusion criteria were the absence of fracture or dislocation in the spinal column, extremities, or pelvis. Patients with solitary cervical spine fractures, solitary penetrating injuries of the neck, or solitary hand fractures were also excluded, as these patients are treated in non-orthopaedic wards at HUH. A total of 175 patients were included in this study. Data were retrieved from the local NTR database and from the patients’ electronic hospital medical records.

From the NTR we retrieved the patient’s identification number, sex, age, preinjury physical status as defined by the American Society of Anesthesiologists (ASA PS classification) [18], ISS, NISS, mechanism of injury (MOI), whether the traumatic event was work related, highest level of treatment, length of stay (LOS) in the intensive care unit (ICU) and in-hospital LOS. Further, information about AIS codes, mortality at day 30, and, when available, cause of death were analysed.

From the patient records, we retrieved information about the main injury, number of orthopaedic injuries, localisation of the orthopaedic injuries, open vs. closed orthopaedic injury, whether the patient received operative treatment for orthopaedic injuries, and, if so, location, type of operative treatment, and time until surgery.

The patients were divided into two age groups: Group 1 (age < 65 years) and Group 2 (age ≥ 65 years). Data on the age distribution of the population in Western Norway for the study period were obtained from Statistics Norway [19].

All fractures and dislocated joints treated by the orthopaedic surgeons at HUH, were registered as orthopaedic injuries. Segmental fractures were classified as one fracture. However, if a long bone had a proximal and a distal fracture with a spared segment in between, this was recorded as two injuries if the fractures required two different types of treatment. Fractures involving connecting segments of the spine were recorded as a single injury. However, two spinal fractures separated by an uninjured segment, were considered as two injuries. Fractures involving both the radius and ulna (antebrachium fracture) were recorded as one injury, as were lower leg fractures (crus fracture), except when the fractures were located at different levels. If a patient had more than one fracture of the pelvis, it was recorded as a single injury. The sacrum was considered part of the pelvis and not the spine. Crush injury of the foot or fractures involving connecting bones in the foot, were recorded as single injuries. Solitary fractures and dislocations in the cervical spine, hand, ribcage, and head, were not considered orthopaedic injuries as they are treated by other medical specialities than the orthopaedic surgeons at HUH.

Operative treatment included both surgery and closed reduction of dislocated bones where anaesthetic procedures were employed. Some patients came to the hospital in the final hours of the day and received operative treatment the same night (< 12 h after admission). These instances were recorded as 0 days to operation, even if the actual date on which the procedure took place was one calendar day after the hospital admission date. The term “Not an option” under “External fixation” implies that the injury could not be treated with external fixation, e.g. fracture in the spinal column.

In the AIS classification system different injuries have sometimes the same severity score. Thus, to register the injury with the most severe impact on patient outcome we used discharge codes, according to the 10th version of the International Statistical Classification of Diseases and Related Health Problems (ICD-10), in an attempt to grade injuries with the same AIS severity score. The injury considered most severe based on ICD-10 codes in the patient´s medical journal was registered as the main injury. These were categorised as either injury to the ‘thorax’, ‘head’, ‘pelvis’, ‘abdomen’, ‘spinal column’, ‘extremities’, ‘neck’, or ‘burns’.

In the NTR, traffic-related injuries involving pedestrians, bicycles, motorcycles, cars, and other means of transportation are coded in different categories. These were all registered as ‘traffic-related’ in this study. The categories ‘firearm injury’ and ‘penetration by sharp or pointy object’ were both coded as ‘penetrating’. Other categories comprised ‘high-energy fall (HEF)’ (fall from a height > 1 m) and ‘low-energy fall (LEF)’ (fall from the standing position, or a height < 1 m), ‘hit by blunt object’, ‘explosion’, and ‘other’. None of our patients sustained injuries caused by an explosion, hence this category is not presented in the tables.

“Highest level of treatment” is a parameter registered in NTR to measure resource demands. The intensive care unit is considered to be the highest treatment level, followed by postoperative care unit, operating room, ward and emergency department as the lowest treatment level.

### Statistical analysis

Data are shown as numbers (n) and proportions (%); means and standard deviations (SD) for continuous variables; and medians and interquartile ranges (IQR) for categorical variables. Categorical variables were analysed using the Pearson’s chi-squared test or Mann–Whitney U test, and the independent samples t-test was used to analyse continuous variables. A *P*-value < 0.05 (two-tailed) was considered statistically significant. Data were analysed using IBM SPSS, version 26 (IBM Corp., Armonk, New York, USA).

## Results

**Fig. 1:**
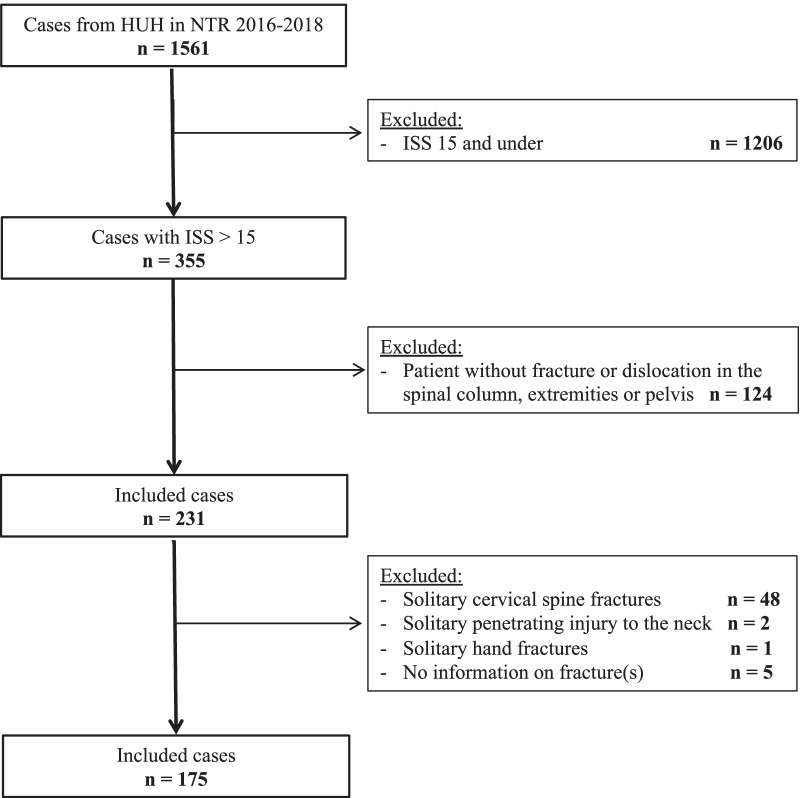
Selection of patients for this study from the Norwegian Trauma Registry (NTR). All subjects were admitted to Haukeland University Hospital during the period 2016–2018. *ISS* injury severity score

Figure 1 shows the selection of the 175 patients who met the inclusion criteria. Baseline characteristics for the age groups and the total number of cases are presented in Table 1.

**Table 1:** Baseline characteristics

Variable	Group 1, age < 65 years	Group 2, age ≥ 65 years	*P* value	Total
	n = 128 (73%)	n = 47 (27%)		(n = 175)
Male (%)	104 (81%)	38 (81%)	1.00^#^	142 (81%)
Age				
Mean (SD)	39.5 (15.2)	73.3 (6.2)		48.6 (20.1)
Median (IQR)	41 (25–53)	72 (68–77)		50 (29–66)
Range	4–64	65–91		4–91
ASA PS classification				
Median (IQR)	1 (1–2)	2 (1–2.5)	< 0.01^+^	1 (1–2)
ASA 1	84 (66%)	14 (30%)		98 (56%)
ASA 2	27 (21%)	20 (43%)		47 (27%)
ASA 3	5 (4%)	11 (23%)		16 (9%)
Unknown	12 (9%)	2 (4%)		14 (8%)
ISS				
Mean (SD)	25.8 (9.4)	24 (7.8)	0.24*	25.3 (9.0)
Median (IQR)	22 (18–30)	22 (17–29)	0.27^+^	22 (17–29)
Range	16–57	17–51		16–57
NISS				
Mean (SD)	30 (12.1)	27 (8.6)	0.07*	29.2 (11.3)
Median (IQR)	27 (22–34)	27 (21–33)	0.29^+^	27 (22–34)
Range	16–75	17–54		16–75
Mechanism of injury			< 0.01^#^	
Traffic-related	68 (53%)	19 (40%)		87 (50%)
High-energy fall	46 (36%)	18 (38%)		64 (37%)
Low-energy fall	0	7 (15%)		7 (4%)
Hit by blunt object	9 (7%)	1 (2%)		10 (6%)
Penetrating	1 (1%)	1 (2%)		2 (1%)
Other	4 (3%)	1 (2%)		5 (3%)
Work related injury	17 (13%)	4 (9%)	0.66^#^	21 (12%)
Unknown	2 (2%)	1 (2%)		3 (2%)
Location of main injury			< 0.01^#^	
Thorax	29 (23%)	21 (45%)		50 (29%)
Head	25 (20%)	10 (21%)		35 (20%)
Pelvis	17 (13%)	5 (11%)		22 (13%)
Abdomen	21 (16%)	0		21 (12%)
Spinal column	14 (11%)	5 (11%)		19 (11%)
Extremities	15 (12%)	3 (6%)		18 (10%)
Neck	4 (3%)	3 (6%)		7 (4%)
Burns	3 (2%)	0		3 (2%)
Orthopaedic injuries				
Mean (SD)	2.3 (1.4)	2.1 (1.0)	0.29*	2.3 (1.3)
Median (IQR)	2 (1–3)	2 (1–3)	0.88^+^	2 (1–3)
Range	1–7	1–4		1–7
Location of orthopaedic injury			0.11^#^	
Spinal column	78 (26%)	33 (33%)		111 (28%)
Pelvis	38 (13%)	16 (16%)		54 (14%)
Upper extremity	82 (28%)	30 (30%)		112 (28%)
Lower extremity	97 (33%)	20 (20%)		117 (30%)
Open injury	23 (18%)	3 (6%)	0.06^#^	26 (15%)
Operative treatment of orthopaedic injury	95 (74%)	25 (53%)	0.01^#^	120 (69%)
External fixation	27 (28%)	5 (20%)	0.60^#^	32 (27%)
Not an option	26 (27%)	9 (36%)		35 (29%)
Days to operative treatment at HUH^a)^				
Mean (SD)	1.99 (3.9)	2.32 (3.5)	0.70*	2.06 (3.8)
Median (IQR)	0 (0–2)	1 (0–3.5)	0.24^+^	0.5 (0–3)
Range	0–20	0–16		0–20
Highest level of treatment			0.96^#^	
Emergency department	2 (2%)	1 (2%)		3 (2%)
Ward	7 (6%)	2 (4%)		9 (5%)
Operating room	3 (2%)	2 (4%)		5 (3%)
Postoperative care unit	55 (43%)	19 (40%)		74 (42%)
Intensive care unit	61 (48%)	23 (49%)		84 (48%)
LOS in ICU				
Mean (SD)	3.4 (6.7)	3.9 (5.6)	0.64*	3.5 (6.4)
Median (IQR)	0 (0–5)	2 (0–7)	0.40^+^	0 (0–5)
Range	0–57	0–23		0–57
LOS in hospital^b)^				
Mean (SD)	15.2 (14.1)	14.2 (13.1)	0.68*	14.9 (13.8)
Median (IQR)	11 (8–18)	13 (6–16)	0.69^+^	11.5 (8–17)
Range	1–109	1–84		1–109
Mortality after 30 days	4 (3%)	7 (15%)	< 0.01^#^	11 (6%)

Baseline characteristics of all patients admitted to HUH with an ISS > 15 and an orthopaedic injury during the period 2016–2018

Missing data for Groups 1 and 2: (a) Days to operative treatment at HUH, 4% and 0%. (b) LOS in hospital, 1% and 4%

*ASA PS classification* American Society of Anesthesiologists physical status classification, *ISS* injury severity score, *NISS* new injury severity score, *HUH* Haukeland University Hospital, *LOS in ICU* length of stay in the intensive care unit, *SD* standard deviation, *IQR* interquartile range

^*^Independent samples *t*-test

^+^Mann–Whitney U test

^#^Pearson´s chi-square test

**Fig. 2:**
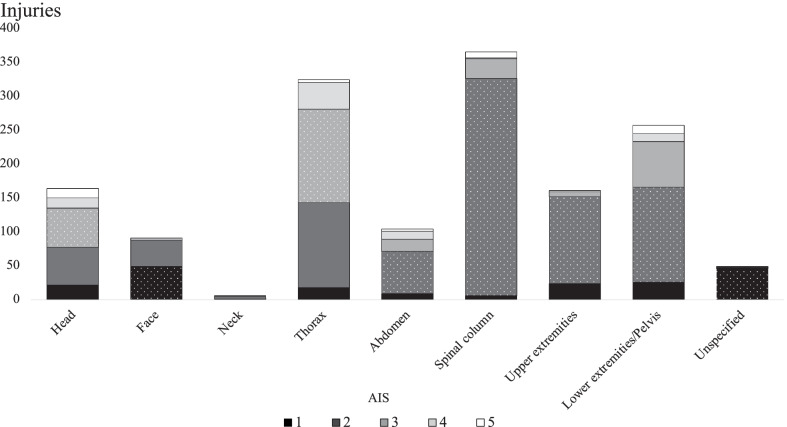
The distribution of AIS scores in relation to the different types of injuries based on the AIS classification system for all patients admitted to Haukeland University Hospital with an ISS > 15, and an orthopaedic injury during the period 2016–2018. Areas with white dots represent the median AIS score. *AIS* abbreviated injury scale, *ISS* injury severity score

Figure 2 shows the distribution of severity scores based on the AIS classification system, in relation to the various types of injuries. When considering injuries with AIS ≥ 3, the most frequently injured body region was the thorax in both groups. This was followed by the lower extremities/pelvis in Group 1, and the head in Group 2.

The proportion of patients who underwent operative treatment decreased as the ASA score increased: 72%, 62%, and 50% of the patients with ASA scores of 1, 2, and 3, respectively. Most patients underwent surgery at HUH. There were 15 (13%) patients who received surgical treatment at other hospitals, either prior to (n = 10) or after (n = 5) admittance to HUH.

The patients who underwent surgery for their orthopaedic injuries had a higher mean ISS and NISS compared to those not receiving operative treatment (ISS; 26 vs. 23, NISS; 30 vs. 27, *p* < 0.05). However, when analysing median ISS and NISS, the differences in severity scores were not significant.

Of the 175 included, 11 (6%) patients succumbed to their injuries within 30 days after injury. The majority of the patients who died were classified as ASA 1 (n = 7). The deceased had a significantly higher mean ISS and NISS than the survivors (ISS; 37 vs. 25, NISS; 42 vs. 28, *p* < 0.01). Both the survivors and non-survivors suffered the same mean number (2.3) of orthopaedic injuries. One patient died of causes related to the injuries, more than 30 days after the trauma.

When looking at the whole patient population most deaths were caused by traffic accidents (64%), and head injury was the most common main injury (45%). In Group 2 most deaths were traffic-related (n = 5). HEFs and traffic-related incidents were the injury mechanisms causing fatalities in Group 1, both being the cause of two deaths. In Group 1 abdominal injuries resulted in most deaths (n = 3), while head injuries was the primary reason for deaths in Group 2 (n = 4).

## Discussion

This study comprised 175 polytrauma patients with orthopaedic injuries, constituting 49% of the 355 trauma patients with an ISS > 15 admitted to HUH during the study period. This corresponds to the Major Trauma Outcome Study reporting that nearly half of the patients suffered musculoskeletal injuries [1].

Traumatic injury previously afflicted mainly young males. However, the proportion of older trauma patients is increasing [7]. Patients aged ≥ 65 years constituted 27% of our study cohort, but only 15% of the population of Western Norway during the study period [19]. This corresponds with the findings of Cuevas-Østrem et al. who reported that this group of patients constituted 33% of their study cohort, but only 17% of the Norwegian population [9].

Both age groups had similar ISS and NISS, comparable to the findings in a similar registry based German study [20]. However, Cuevas-Østrem et al. found a larger percentage of patients with an NISS ≥ 15 among patients aged ≥ 65 years than in those aged 16–64 years [9].

Traffic-related incidents was the dominating injury mechanism in both age groups, while others have reported LEFs as the most prevalent (40%) MOI among patients aged ≥ 65 years [9]. Among trauma patients with an NISS ≥ 9, HEFs were the dominant (28%) MOI in the 16–64 years group [9]. Kocuvan et al. reported that the most frequent MOI in major trauma patients aged < 65 and ≥ 65 years were traffic accidents (52%) and falls (48%), respectively [20].

Our results indicate that LEFs cause more severe injuries in older persons, possibly because the elderly suffer a greater number of injuries to the head, pelvis, femur, and spine [21]. As the bone resilience changes owing to the ageing process, the risk of fractures increases [22]. This has been shown in Scandinavia, where a higher incidence of hip fractures are seen related to osteoporosis [23]. Simultaneously, the risk of falling may increase due to e.g. visual impairment, gait challenges, or the use of various medications [24]. These factors likely contribute to falls being reported as the main cause of trauma-related death among older persons, and are associated with a high rate of head and orthopaedic injuries [24].

Based on ICD-10 coding, thoracic injury was coded as the most frequent main injury in both Group 1 (23%) and 2 (45%), followed by head injury (20% and 21%, respectively). This correlates with the findings from the NTR, using the AIS classification system, where the “thorax” and “head” had the highest AIS scores (median AIS score = 3). Kocuvan et al. reported trauma to the head (55%) and thorax (55%) as the most common injuries among patients aged ≥ 65 years; however, the younger patients mainly sustained head (52%) and musculoskeletal injuries (46%) [20].

Despite suffering the same number of orthopaedic injuries, a significantly higher proportion of patients in Group 1 (74%) received operative treatment for their orthopaedic injuries compared to patients in Group 2 (53%; *p* = 0.01). There could be several  explanations for this difference, such as higher preinjury ASA score or perhaps the elderly suffering fractures more suited to conservative treatment due to low-energy injury mechanisms. Our study did not include data permitting conclusions about what causes these differences. Further exploration of the causes behind treatment variations are warranted to ensure optimal trauma care to all patients irrespective of age and general health.

In accordance with the high ISS, nearly half of the patients in both age groups were treated in the ICU. This differs from studies reporting a lower ICU admission rate among trauma patients aged ≥ 65 years [21, 25, 26]. However, other investigators have found that older trauma patients have a higher ICU admittance rate [21]. There is a non-significant trend that Group 2 patients stayed longer, both in the ICU and in hospital, compared to Group 1.

Eleven (6%) of the 175 patients in our study died within 30 days after injury. This is lower than reported in comparable studies [5, 27]. We found that the older patients had higher mortality risk (15%) than younger patients (3%; *p* < 0.01), although displaying similar ISS and NISS. This corresponds to several studies reporting higher mortality among older patients [6, 9, 21, 28–30].

Most deaths were traffic-related (64%), and head injury was the most common main injury (45%) as previously reported [31, 32]. However, fall from height has been reported as the most common MOI in trauma-related death [33]. Injury to the central nervous system has been identified as the dominant cause of death in polytrauma patients [31, 33, 34]. Traumatic brain injury (TBI) has previously been reported as the leading cause of death in the Norwegian geriatric trauma population [6]. In the elderly, TBI is most frequently caused by falls from an upright position [35]. Patients with low-energy MOI may not be received by a trauma team, increasing the risk of under-triage and delays of time-critical treatment [15]. Early surgical management and timely rehabilitation result in better outcomes for elderly suffering TBI [35]. To minimise the risk of under-triage of the elderly, it has been suggested that high age alone should be a criterion for TTA [15]. This is not current practice in Norway.

The longer ICU and hospital stays, and the higher mortality rate among patients aged ≥ 65 years, are likely impacted by pre-existing chronic conditions and frailty, increasing susceptibility to medical complications [30]. To compensate for these factors, Advanced Trauma Life Support and the Eastern Association for the Surgery of Trauma Geriatric Trauma Guidelines have recommended a prompt and aggressive approach, as this has shown to reduce the mortality rate among older trauma patients [7]. Moreover, this reduces the negative impact of post-injury complications on outcomes [30]. Other investigators have found that use of a geriatric consult service may lead to better outcomes and more efficient in-hospital treatment [36].

This is a descriptive study, which gives an overview of characteristics of our patient population. We have found differences between the age groups, but our data is not sufficient to draw any conclusions about what causes these differences. Future studies investigating reasons behind the variation in treatment and how the different therapy strategies impact patient outcomes are warranted. This will hopefully help to further develop strategies that could improve outcome for the elderly polytrauma patient.

Our study does not provide information about the rehabilitation process or other outcomes than mortality. It would be interesting to do a follow-up study of severely injured trauma patients, examining the rehabilitation required, discharge destination, functional outcome, and quality of life.

There are several limitations to this study. The study population was relatively small due to the short study period, low trauma volume, and patients not meeting the inclusion criteria. The limited number of patients resulted in less power for statistical comparisons. The patients included were from a single trauma centre, and thus may have been influenced by regional therapeutic traditions and patient characteristics. Although the personnel performing registration in NTR are certified as such [37], inter-observer variability cannot be ruled out. The retrospective design also prevents exploration of causal relationships, and may only identify associations.

## Conclusions

Even with similar ISS and NISS, the mortality was significantly higher among patients aged ≥ 65 years compared to those aged < 65 years. Younger patients more frequently received operative treatment for their orthopaedic injuries than the elderly. There may be multiple reasons for this difference, but our study does not have sufficient data to draw conclusions as to why. Future studies may provide a deeper understanding of what causes treatment variation between age groups, which would hopefully help to further develop strategies to improve outcome for the elderly polytrauma patient.

## Data Availability

The datasets generated and/or analyzed during the current study are not publicly available due to it being part of the local trauma registry but are available from the corresponding author on reasonable request.

## Abbreviations

NTR: Norwegian Trauma Registry
AIS: Abbreviated Injury Scale
ISS: Injury Severity Score
NISS: New Injury Severity Score
TTA: Trauma team activation
HUH: Haukeland University Hospital
TC: Trauma Center
ASA PS classification: Preinjury physical status as defined by the American Society of Anesthesiologists
MOI: Mechanism of injury
LOS: Length of stay
ICU: Intensive care unit
ICD-10: The 10th Version of the International Statistical Classification of Diseases and Related Health Problems
HEF: High-energy fall
LEF: Low-energy fall
SD: Standard deviation
IQR: Interquartile range
TBI: Traumatic brain injury

## References

[1] Bach JA, Leskovan JJ, Scharschmidt T, Boulger C, Papadimos TJ, Russell S (2017). The right team at the right time - Multidisciplinary approach to multi-trauma patient with orthopedic injuries. Int J Crit Illn Inj Sci.

[2] Balogh ZJ, Reumann MK, Gruen RL, Mayer-Kuckuk P, Schuetz MA, Harris IA (2012). Advances and future directions for management of trauma patients with musculoskeletal injuries. Lancet.

[3] Gomberg BF, Gruen GS, Smith WR, Spott M (1999). Outcomes in acute orthopaedic trauma: a review of 130,506 patients by age. Injury.

[4] Wennergren D, Möller M (2018). Implementation of the Swedish Fracture Register. Unfallchirurg.

[5] Brinck T, Handolin L, Paffrath T, Lefering R (2015). Trauma registry comparison: six-year results in trauma care in Southern Finland and Germany. Eur J Trauma Emerg Surg.

[6] Ringen AH, Gaski IA, Rustad H, Skaga NO, Gaarder C, Naess PA (2019). Improvement in geriatric trauma outcomes in an evolving trauma system. Trauma Surgery & Acute Care Open.

[7] Cuevas-Østrem M, Røise O, Wisborg T, Jeppesen E (2020). Geriatric trauma—a rising tide. Assessing patient safety challenges in a vulnerable population using Norwegian Trauma Registry Data and Focus Group Interviews: protocol for a mixed methods study. JMIR Res Protoc..

[8] Chow J, Kuza CM (2022). Predicting mortality in elderly trauma patients: a review of the current literature. Curr Opin Anaesthesiol..

[9] Cuevas-Østrem M, Røise O, Wisborg T, Jeppesen E (2021). Epidemiology of geriatric trauma patients in Norway: a nationwide analysis of Norwegian Trauma Registry data, 2015–2018. Retrosp Cohort Study Injury.

[10] Dahlhaug M, Røise O. Nasjonalt traumeregister; årsrapport for 2019 med plan for forbedringstiltak (in Norwegian). Oslo University hospital HF; 2020. Available from: https://www.kvalitetsregistre.no/sites/default/files/2021-02/%C3%85rsrapport%202019%20Nasjonalt%20traumeregister.pdf

[11] Ringdal KG, Coats TJ, Lefering R, Di Bartolomeo S, Steen PA, Røise O (2008). The Utstein template for uniform reporting of data following major trauma: a joint revision by SCANTEM, TARN, DGU-TR and RITG. Scand J Trauma Resusc Emerg Med.

[12] Gennarelli TA, Wodzin E. The Abbreviated Injury Scale. Association for the Advancement of Automotive Medicine, Barrington, IL (2005) Update 2008.

[13] Baker SP, O'Neill B, Haddon W, Long WB (1974). The injury severity score: a method for describing patients with multiple injuries and evaluating emergency care. J Trauma.

[14] Osler T, Baker SP, Long W (1997). A modification of the injury severity score that both improves accuracy and simplifies scoring. J Trauma..

[15] Nordgarden T, Odland P, Guttormsen AB, Ugelvik KS (2018). Undertriage of major trauma patients at a university hospital: a retrospective cohort study. Scand J Trauma Resusc Emerg Med.

[16] Osteras O, Brattebo G, Heltne JK (2016). Helicopter-based emergency medical services for a sparsely populated region: a study of 42,500 dispatches. Acta Anaesthesiol Scand.

[17] Palmer C (2007). Major trauma and the injury severity score—Where should we set the bar?. Annu Proc Assoc Adv Automot Med.

[18] ASA Physical Status Classification System American Society of Anesthesiologists; 2019 [updated 2020, December 13. Available from: https://www.asahq.org/standards-and-guidelines/asa-physical-status-classification-system.

[19] The Population in Norway 2021: Statistics Norway; 2021. Available from: https://www.ssb.no/en/befolkning/statistikker/folkemengde/aar-per-1-januar.

[20] Kocuvan S, Brilej D, Stropnik D, Lefering R, Komadina R (2016). Evaluation of major trauma in elderly patients—a single trauma center analysis. Wien Klin Wochenschr.

[21] Hildebrand F, Pape HC, Horst K, Andruszkow H, Kobbe P, Simon TP (2016). Impact of age on the clinical outcomes of major trauma. Eur J Trauma Emerg Surg.

[22] Ettinger MP (2003). Aging bone and osteoporosis: strategies for preventing fractures in the elderly. Arch Intern Med.

[23] Kanis JA, Oden A, McCloskey EV, Johansson H, Wahl DA, Cooper C (2012). A systematic review of hip fracture incidence and probability of fracture worldwide. Osteoporos Int.

[24] Evans D, Pester J, Vera L, Jeanmonod D, Jeanmonod R (2015). Elderly fall patients triaged to the trauma bay: age, injury patterns, and mortality risk. Am J Emerg Med.

[25] Taylor MD, Tracy JK, Meyer W, Pasquale M, Napolitano LM (2002). Trauma in the elderly: intensive care unit resource use and outcome. J Trauma.

[26] Burstow M, Civil I, Hsee L (2019). Trauma in the elderly: demographic trends (1995–2014) in a Major New Zealand Trauma Centre. World J Surg.

[27] Schwartz AM, Staley CA, Wilson JM, Reisman WM, Schenker ML (2020). High acuity polytrauma centers in orthopaedic trauma: Decreasing patient mortality with effective resource utilization. Injury.

[28] Mitra B, Cameron PA (2012). Optimising management of the elderly trauma patient. Injury.

[29] Keller JM, Sciadini MF, Sinclair E, O'Toole RV (2012). Geriatric trauma: demographics, injuries, and mortality. J Orthop Trauma.

[30] Perdue PW, Watts DD, Kaufmann CR, Trask AL (1998). Differences in mortality between elderly and younger adult trauma patients: geriatric status increases risk of delayed death. J Trauma.

[31] de Knegt C, Meylaerts SA, Leenen LP (2008). Applicability of the trimodal distribution of trauma deaths in a Level I trauma centre in the Netherlands with a population of mainly blunt trauma. Injury.

[32] Chiara O, Scott JD, Cimbanassi S, Marini A, Zoia R, Rodriguez A (2002). Trauma deaths in an Italian urban area: an audit of pre-hospital and in-hospital trauma care. Injury.

[33] El Mestoui Z, Jalalzadeh H, Giannakopoulos GF, Zuidema WP (2017). Incidence and etiology of mortality in polytrauma patients in a Dutch level I trauma center. Eur J Emerg Med.

[34] Pfeifer R, Tarkin IS, Rocos B, Pape HC (2009). Patterns of mortality and causes of death in polytrauma patients—Has anything changed?. Injury.

[35] Ruge T, Carlsson AC, Hellstrom M, Wihlborg P, Undén J (2020). Is medical urgency of elderly patients with traumatic brain injury underestimated by emergency department triage?. Ups J Med Sci.

[36] Brooks SE, Peetz AB (2017). Evidence-based care of geriatric trauma patients. Surg Clin N Am.

[37] Dahlhaug M, Røise O. Nasjonalt traumeregister; årsrapport for 2020 med plan for forbedringstiltak (in Norwegian). Oslo University Hospital HF; 2021. Available from: https://nkt-traume.no/wp-content/uploads/2021/09/Arsrapport_NTR_2020.pdf

